# Development, Characterization and Cell Viability Inhibition of PVA Spheres Loaded with Doxorubicin and 4′-Amino-1-Naphthyl-Chalcone (D14) for Osteosarcoma

**DOI:** 10.3390/polym13162611

**Published:** 2021-08-06

**Authors:** Viviane Seba, Gabriel Goetten de Lima, Bruno L. Pereira, Gabriel Silva, Luiza Steffens Reinhardt, Pablo Ricardo Arantes, Bor Shin Chee, Mariana Bastos dos Santos, Suzelei C. França, Luis Octavio Regasini, Ana Lúcia Fachin, Zhi Cao, Michael J. D. Nugent, Mozart Marins

**Affiliations:** 1 Materials Research Institute, Athlone Institute of Technology, Athlone, Co. Westmeath, Ireland; vivianeseba@gmail.com (V.S.); ggoetten@research.ait.ie (G.G.d.L.); b.schee@research.ait.ie (B.S.C.); zcao@research.ait.ie (Z.C.); 2 Biotechnology Unit, University of Ribeirão Preto, Ribeirão Preto 14096-900, SP, Brazil; sfranca@unaerp.br (S.C.F.); afachin@unaerp.br (A.L.F.); 3 Post Graduate Program in Engineering and Materials Science—PIPE, Federal University of Paraná, Curitiba 81530-000, PR, Brazil; brnlp7@gmail.com; 4 School of Pharmaceutical Sciences of Ribeirão Preto, University of São Paulo, Ribeirão Preto 14040-900, SP, Brazil; biel-189@hotmail.com; 5 Laboratory of Genetic Toxicology, Federal University of Health Sciences of Porto Alegre, Porto Alegre 90050-170, RS, Brazil; luizasteffens@live.com; 6 School of Biomedical Sciences and Pharmacy, Hunter Medical, College of Health, Medicine and Wellbeing, University of Newcastle, Newcastle, NSW 2308, Australia; 7 Department of Bioengineering, University of California, Riverside, CA 92507, USA; pabloa@ucr.edu; 8 Laboratory of Green and Medicinal Chemistry, Department of Chemistry and Environmental Sciences, Institute of Biosciences, Humanities and Exact Sciences, São Paulo State University (UNESP), São José do Rio Preto 15054-000, SP, Brazil; mariana19bsantos@gmail.com (M.B.d.S.); luisregasini@gmail.com (L.O.R.); 9 Medicine School, University of Ribeirão Preto, Ribeirão Preto 14096-900, SP, Brazil; 10 Pharmaceutical Sciences School, University of Ribeirão Preto, Ribeirão Preto 14096-900, SP, Brazil

**Keywords:** osteosarcoma, chalcone, doxorubicin, PVA, freeze–thaw

## Abstract

Chalcones (1,3-diaryl-2-propen-1-ones) are naturally occurring polyphenols with known anticancer activity against a variety of tumor cell lines, including osteosarcoma (OS). In this paper, we present the preparation and characterization of spheres (~2 mm) from polyvinyl alcohol (PVA) containing a combination of 4′-Amino-1-Naphthyl-Chalcone (D14) and doxorubicin, to act as a new polymeric dual-drug anticancer delivery. D14 is a potent inhibitor of osteosarcoma progression and, when combined with doxorubicin, presents a synergetic effect; hence, physically crosslinked PVA spheres loaded with D14 and doxorubicin were prepared using liquid nitrogen and six freeze–thawing cycles. Physical-chemical characterization using a scanning electron microscope (SEM), differential scanning calorimetry (DSC), Fourier transform infrared spectroscopy (FTIR) and X-ray diffraction (XRD) presented that the drugs were incorporated into the spheres via weak interactions between the drugs and the polymeric chains, resulting in overall good drug stability. The cytotoxicity activity of the PVA spheres co-encapsulating both drugs was tested against the U2OS human osteosarcoma cell line by 3- (4,5-dimethylthiazol-2-yl) -2,5-diphenyltetrazolium bromide (MTT) assay, and compared to the spheres carrying either D14 or doxorubicin alone. The co-delivery showed a cytotoxic effect 2.6-fold greater than doxorubicin alone, revealing a significant synergistic effect with a coefficient of drug interaction (CDI) of 0.49. The obtained results suggest this developed PVA sphere as a potential dual-drug delivery system that could be used for the prominent synergistic anticancer activity of co-delivering D14 and doxorubicin, providing a new potential strategy for improved osteosarcoma treatment.

## 1. Introduction

Chalcones are phytocompounds found abundantly in nature and are precursors of different types of flavonoids, such as flavonols, catechins, isoflavones, and others. These compounds are characterized by the presence of two aromatic rings bounded by an α,β-unsaturated ketone that forms an oxygenated heterocycle [1]. Several studies suggest that chalcones, and their derivatives, present antitumor activities via induction of cell cycle arrest and/or apoptosis, along with the inhibition of angiogenesis and metastasis in different cancer cells [2,3,4,5]. Recently, our group demonstrated in osteosarcoma (a highly metastatic cancer) that synthetic chalcone 4′-Amino-1-Naphthyl-Chalcone (D14) can upregulate the p53 expression that inhibits migration and invasion of osteosarcoma cancer cells [6], corresponding to fundamental processes for metastasis development and progression. In addition to their isolated effects, chalcones have synergistic effects when associated with classical antitumor drugs, such as doxorubicin (doxo), etoposide and cisplatin [7,8,9]. Despite this proven anticancer property, usage of these compounds in the therapeutic field is limited. This is due to some inherited properties, such as high hydrophobicity, low bioavailability and poor stability in physiological solutions [10,11]. In recent years, drug delivery systems (DDS) have been suggested in order to encapsulate these compounds into carriers that improve drug solubility, bioavailability, and its crucial therapeutic efficiency [12,13,14].

Polyvinyl alcohol (PVA) is a water-soluble synthetic polymer that exhibits remarkable properties, such as a lack of toxicity, high biocompatibility, biodegradability, high swelling ratio, thermal stability, suitable chemical resistance and high hydrophilicity [15,16]. Furthermore, PVA’s major functional group is composed of hydroxyl groups that indicate a strong chemical resistance while also relating to its blending ability to other synthetic and natural polymers [17,18,19,20]. As a result of these features, PVA has been used for drug delivery and tissue scaffolds [15,21,22,23,24,25]. Microspheres and hydrogels based on PVA can be prepared by subjecting PVA solution to repeated freeze–thaw cycles, where physical crosslinking occurs through hydrogen bonding based on ice crystal growth [11,26]. This method has the benefit of not leaving residual solvent that can increase toxicity [26].

Polymeric spheres have been widely used in pharmaceutical and biomedical applications [27], while also used as a DDS; these spheres can also function as a therapeutic drug reservoir, due to their controlled and sustained release profiles, which leads to a suitable drug concentration and improved therapeutic outcomes. Moreover, requirements for nano and microspheres used in DDS can be related to their biocompatibility, degradability, stability, and capacity to incorporate drugs without the loss of their bioactivity. Additionally, spheres have been developed for the treatment of several diseases, including cancer, owing to their potential for targeted delivery with active cellular uptake in localized tumors [28,29].

Production of spheres to act as a drug release has been reported before by previous works. In the case of a cryogenic sphere, our group presented that depending on the freezing cycles performed, they can alter the structure and present a good, steady sphere morphology so as to act as an immediate drug release system [30]. Nonetheless, there are now novel developed strategies for nanoparticle production, such as supercritical assisted electrospray [31].

Previous works exhibited the use of PVA-based microparticles and microspheres for doxo delivery [11,32] and entrapment of chalcone-related compounds, such as genistein and trihydroxyisoflavone [33,34]. Moreover, the surface of PVA microparticles can be coated with specific molecules to improve doxo delivery to cancer cells [35]. Recently, a PVA-hydroxyapatite nanocomposite was produced and used to encapsulate doxo aiming at the repair of bone tissues affected by osteosarcoma. Using an osteosarcoma cell line, MG 63, high cytotoxicity was induced by the scaffold [36,37]; however, this was without comparison to the pure drug. Therefore, the suggested DDS may be suitable for patients undergoing surgical procedures, whereas these spheres could deliver doxo without the necessity of invasive procedures.

The significant anticancer effects of chalcones in osteosarcoma cells and the promising properties of PVA as a drug carrier are noteworthy. Therefore, the main goal of this work was aimed at producing PVA spheres containing a mixture of D14 and doxo, to evaluate if this PVA-DDS is able to improve its stability and promote sustained drug release. Primarily, we assessed if these compounds were efficiently encapsulated into PVA spheres and analyzed if encapsulation altered the physicochemical features of these molecules. Finally, a cytotoxicity test was performed to analyze their activity against the human osteosarcoma U2OS cell line.

## 2. Materials and Methods

### 2.1. Cell Culture and Chemicals

The U2OS human osteosarcoma cell line was kindly provided by the Genome Stability Lab of the National University of Ireland (Galway, Ireland). PVA Mw 56–98 (98–98.8% hydrolysis), dimethyl sulfoxide (DMSO), MTT (3- (4,5-dimethylthiazol-2-yl) -2,5-diphenyltetrazolium bromide), penicillin, McCoy’s 5A medium, fetal bovine serum (FBS), and streptomycin were purchased from Sigma-Aldrich^®^ (St. Louis, USA). D14 was synthesized and donated by Luis Octavio Regasini (Department of Chemistry and Environmental Chemistry of the State University of São Paulo, São José do Rio Preto, Brazil). The doxorubicin (hydrochloride) was purchased from Cayman Chemical (Cambridge, UK).

### 2.2. Spheres Production

To produce the spheres, PVA was dissolved (using a hot plate (70 °C)) in deionized water (2% vol) and allowed to stir until a uniform solution was obtained. Then, the temperature was lowered (40 °C) and 10 mg of D14 was diluted in 100 μL of DMSO and added to the PVA solution. Similarly, 2.5 mg of doxo was diluted in water and the solution was added to the PVA and D14 mixture. This solution remained in continuous motion on a hot plate (40 °C) until completely uniform. After this period, with the aid of a 100 μL tip, this solution was placed in liquid nitrogen (~−196 °C) by using the sessile drop technique, which formed the spheres (~2 mm). These spheres were incubated at −80 °C for 1 h, followed by 6 min at room temperature and again for 1 h at −80 °C. This cycle was repeated six times. These spheres were freeze-dried overnight. Additionally, spheres containing only D14, or doxo and control samples, were also prepared for comparison reasons.

### 2.3. Fourier Transform Infrared Spectroscopy (FTIR)

In order to analyze the occurrence of chemical interactions between D14, doxo and PVA, Fourier transform infrared spectroscopy (FTIR) with the PerkinElmer Spectrum One (Waltham, MA, USA) was used. The IR spectra were registered using the spectral range of 4000–500 cm^−1^ with a resolution of 4 cm^−1^ and an arithmetic average of 64 scans was used.

### 2.4. Differential Scanning Calorimetry (DSC)

In order to perform a thermal analysis, differential scanning calorimetry (DSC) equipment was used (TAQ2000 of TA instruments, New Castle, DE, USA). Studied samples were sealed in hermetic aluminum pans (Hermetic Pans, TA Instruments, New Castle, DE, USA) using pre-weighted samples of around 7–10 mg. An empty pan was used as a reference. In any of the samples, the analysis was carried out in a heating ramp mode from 20 °C to 280 °C and a heating rate of 10 °C/min under the flow of nitrogen gas using two samples per condition.

### 2.5. Scanning Electron Microscopy (SEM)

To analyze the spheres that were loaded with D14 and doxo, a scanning electron microscope (SEM) (TESCAN Mira3 Performance in Nanospace, Brno, CZ) in backscattered electron mode (BSE) was used. The analyses were performed on whole samples and cryofractured samples for cross-sectional regions and they were all sputter coated with gold using Baltec SCD 005 for 110 s at 0.1 mBar of vacuum. For recordings of the images, an acceleration voltage of 15 kV with an amplification range of 300–1000 was used.

### 2.6. X-ray Diffraction (XRD)

The radiation used was Cu K-alpha, X-ray wavelength of 1.5406 Å, angular velocity: 0.7 degree/minute and range: 5° to 40°. The X-ray diffraction (XRD) equipment used was Shimadzu 7000 (Shimadzu, Duisburg, Germany) coupled with a monochromator. In search of better results, a special aluminum support was made. Deconvolution of the PVA main peak was performed (12–34 (2θ°)) using the Fityk software (v.1.3.1, Marcin Wojdyr, open source software available at http://fityk.nieto.pl) by first performing a baseline correction. The Levenberg–Marquardt algorithm was used to fit a Gaussian function in order to obtain the amorphous and crystallinity peaks using many interactions until a better fit was not found by the software.

### 2.7. Molecular Docking Studies

Docking calculations were performed with AutoDock Vina (v.1.2.1, Scripps Research, San Diego, CA, USA) [38]. The structure of PVA was obtained from a previous work [15]. AutoDock Vina operates by performing a conformational search approach that is gradient based. Thus, this method defines the search space by a grid box defined by the coordinates of the center of the box and its dimensions of x, y, and z in the grid resolution internally assigned to 1Å [38]. The number of binding modes was determined at 100 and the exhaustiveness at 200 to control how many times the calculations are repeated. All rotatable dihedral angles of D14 and doxo were treated as flexible. The orientations of D14 and doxo at the binding sites on PVA were selected from docked conformations as representative of the lower energy clusters generated by AutoDock Vina.

### 2.8. Cell Viability Assay

MTT assay was used to verify the viability of the osteosarcoma cell line. U2OS cells were cultured in McCoy’s 5A medium, supplemented with 10% FBS, 100 U/mL penicillin and 100 μg/mL streptomycin. The cells were cultured at 37 °C in a humidified atmosphere of 5% CO_2_. Briefly, U2OS cells were seeded at a concentration of 10,000 cells/well in 96-well culture plates and were held in three biological replicates. Then, the plates were incubated overnight. After that period, the cells were treated (empty PVA spheres, PVA spheres loaded with D14, PVA spheres loaded with doxo and PVA spheres with the drugs in combination) for 24 h and compared to non-treated cells (negative control). After treatment with the spheres, the culture medium was changed to a fresh medium containing MTT (0.5 mg/mL) and the plates were incubated for 3 h at 37 °C. Finally, in order to analyze cell viability, the plates were taken to a microplate reader (BioTek Synergy HT, Swindon, UK) and were quantified by absorbance detection (550 nm). The percentage of cell viability inhibition was determined as follows:

(1) $$\%\;Cell\;Inhibition\;=\;100\;-\;\frac{ABS\;of\;treated\;cells-ABS\;of\;blank\;}{ABS\;of\;non-treated\;cells-ABS\;of\;blank}$$

In addition, the mean of each treatment group was statically compared with the mean of every other group. Based on the MTT results, the coefficient of drug interaction (CDI) was calculated and used to analyze the synergistic effect of drug association (D14 + doxo) [39,40,41]. The CDI was determined as follows:

(2) $$CDI\;=\frac{AB}{\left(A\times B\right)}$$

where *AB* is the ratio of the drug association group (D14 + doxo) to the control group, and *A* or *B* are the ratios of the single drug group (D14 or doxo) to the control group. CDI values of 1, <1 or >1 express additive, synergistic or antagonistic effects, respectively. A CDI value < 0.7 demonstrates a strong synergism of the drug association.

### 2.9. Statistical Analysis

Biological experiments were executed at least in triplicate. Quantitative data were shown as mean and standard deviation. A statistical analysis, in which the data followed parametric data, was performed by using one-way ANOVA followed by a Tukey post hoc test using GraphPad Prism 5 software (La Jolla, CA, USA). Significant results were considered when *p* < 0.05.

## 3. Results and Discussion

In previous studies, we characterized the antimetastatic activity of D14 against osteosarcoma cell lines, which presented reduced cell migration and invasion, while promoting p53 protein expression and a decrease in matrix metalloproteinases [6]. In this study, we developed PVA spheres containing doxo and D14 as co-delivery, aiming at increasing the effectiveness of doxo treatment against osteosarcoma cells.

The freeze–thaw cycles on PVA allowed us to physically crosslink the structure and produce a hydrogel that presented a sphere morphology simultaneously loaded with a hydrophobic drug (D14) and a hydrophilic drug (doxo), devoid of carrier requirements or the need for an increase in drug dosages.

**Figure 1:**
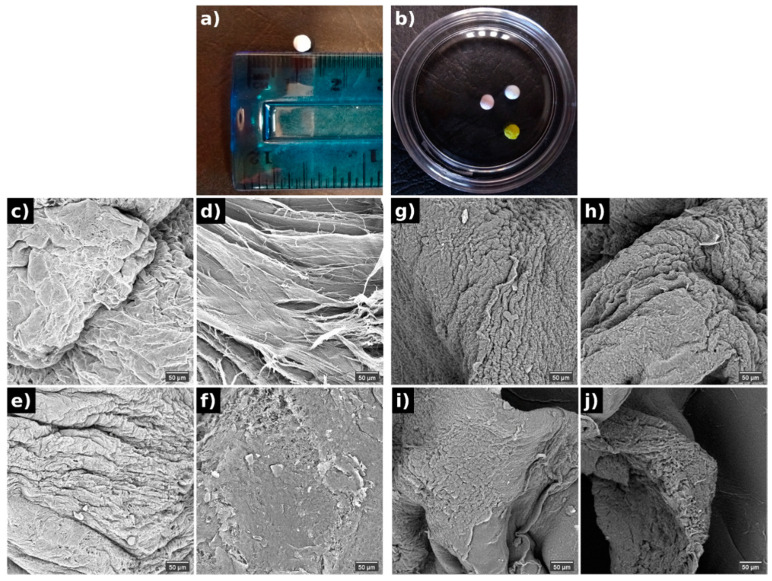
Macroscopic images of the PVA spheres (**a**,**b**) and its morphology evaluated by SEM from the surface (**c**,**e**,**g**,**i**) and internal (**d**,**f**,**h**,**j**) structure of (**c**,**d**) pure PVA, (**e**,**f**) doxo-PVA, (**g**,**h**) D14-PVA, (**i**,**j**) D14-doxo-PVA.

Four different PVA spheres were prepared, and all of them presented a spherical shape with a mean size of 2 mm (Figure 1a,b). SEM images (Figure 1c–j) exhibited that these PVA spheres had porous surfaces with a fibrous appearance, observed in the highest magnification (Figure 1c,e,g,i). The spheres loaded with doxo had a more deformed shape with pronounced cavities, but in larger enlargements, a smoother structure appeared. The spheres containing D14 had a rougher morphology, which may facilitate the diffusion of the compound, whereas the spheres with the association of D14 and doxo displayed a homogeneous structure. This internal porosity (Figure 1d,f,h,j) and surface morphology might have provided distinctive release properties to the dual-drug delivery system (DDS). Overall, the suggested morphology may present distinct drug release profiles as their punctual density/concentration may be higher for specific parts of the polymer since the drying mechanism shrinks the surface parts of the polymer to a more non-conformal state.

**Figure 2:**
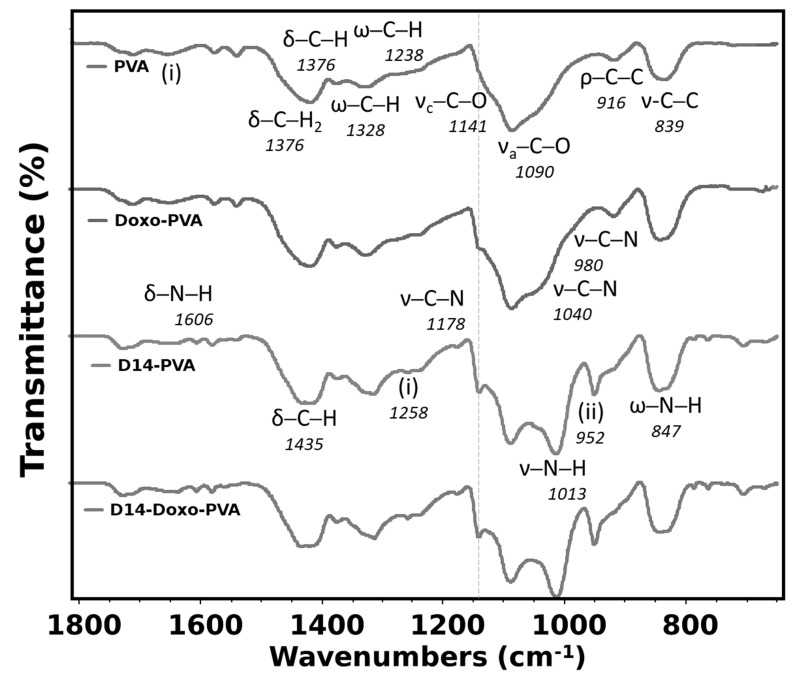
Fourier transform infrared spectroscopy (FTIR) analysis of PVA sphere, doxo-loaded PVA sphere, D14-loaded PVA sphere and D14 and doxo-loaded PVA sphere. In the figure, the symbols represent ν—stretching, δ—bending, ρ—rocking, ω—wagging. Region (i) is water absorption and (ii) represents aromatic ketone—C-CO aryl skeletal vibration.

The FTIR analysis (Figure 2) was used to verify if intramolecular bonding occurred between drugs and polymeric chains of the PVA. The large spectrum region measured for this analysis presents the typical bands of PVA (Appendix A), such as in the range of 3400 cm^−1^ (stretching vibration of the OH groups) and 2900 cm^−1^ (symmetric and asymmetric stretching vibration of C-H). Particularly for this study, the region within 1800–640 was analyzed and related to the most significant differences when the studied drugs were added to the PVA hydrogel. As expected, the most important assigned bands of PVA were perceived and presented a typical spectrum [42]. Contrarily, doxorubicin spectra presented many characteristic peaks (Appendix A) and were difficult to assign for all its bands; although, only a few of its bands were seen when added to the PVA hydrogel as a shoulder to the stretching C-O region. These bands were the 940 and 1040, assigned to the stretching vibration of C-N, and corresponded to different quinone and ketone carbonyl, and are perceived in the spectra because it probably formed an intermolecular bond with the PVA [43]. Previous works also exhibited that interactions between doxo and other materials occurred by the appearance of these bands [44,45].

In the case of D14, few bands are seen for its pure material with the majority being assigned (Appendix A), and this spectrum is formed because of the aromatic rings, amine and aromatic ketone groups found in this compound. When this drug is synthesized with the PVA, many important bands of D14 are seen in this hydrogel, indicating that a good interaction occurred between both materials. More importantly, the bands of N-H stretching vibration of this pure drug blue-shifted to a more specific region of the PVA, which is also assigned to stretching of C-O from the OH groups. Therefore, it is possible that the amine groups may have been modified in the synthesis to crosslink with the PVA in the OH side-group. In addition, there is an even more defined band of the PVA crystallinity, indicating higher-ordered chains. Nonetheless, when both drugs were added to this hydrogel, few differences were perceived in comparison to when only D14 was added. This indicates that the doxorubicin drug may have lower stability, which also relates to a faster drug release since it is weakly bound with PVA. Furthermore, the effect of these two drugs also indicates that the doxo is more loosely bounded in this structure since the shoulder is hardly perceived.

**Figure 3:**
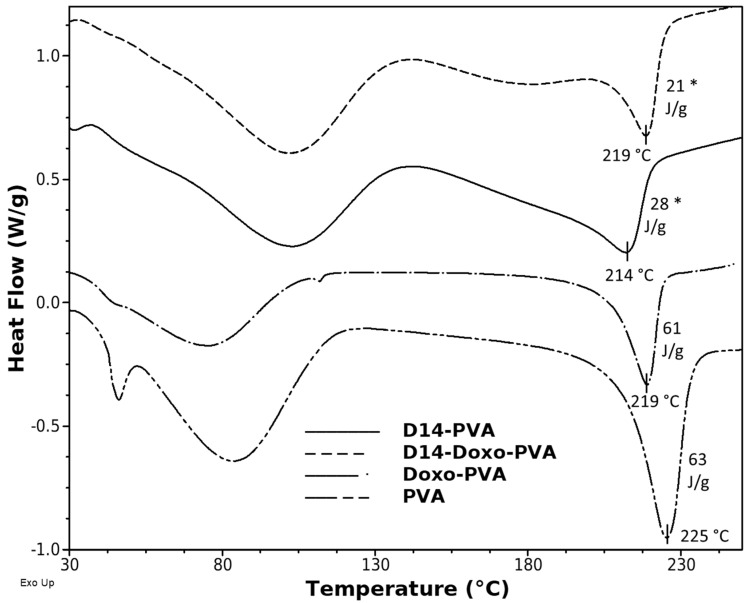
Differential scanning calorimetry thermograms of PVA spheres, D14-loaded PVA sphere, doxo-loaded PVA sphere and D14 and doxo-loaded PVA sphere. The y-axis represents watts/sample g (W/g). A statistically significant difference for D14 group is indicated by (*).

DSC analyses were carried out in order to evaluate the thermal characteristics of the PVA spheres (Figure 3). As shown in Figure 3, the DSC thermogram of PVA spheres displays two main endothermic peaks. The sharp and strong peak around 225 °C corresponds to the melting point of PVA. The broad peak at approximately 85.53 °C can be related to the evaporation of water, as well as the glass transition temperature of the polymer and relaxation of polymeric ordered chains, which were established during the freeze–thaw cycling [46]. The addition of doxo slightly changed the structure by decreasing the Tm and the amount of heat of fusion, meaning that the drug did not heavily affect the crystallinity of the PVA. However, when D14 was incorporated, a heavy decrease in the crystallinity occurred; this can be related to the lowest melting point and smallest heat of fusion of PVA. This result is in agreement with the FTIR interpretation, whereas the addition of D14 led the PVA to bind with the amine groups of this drug and altered the chain orientation and increased its amorphous region [47,48]. However, the peaks moved slightly to lower temperatures and the intensity of the peaks also decreased, suggesting a minor interference in the lattice between the functional groups (OH) of the PVA chains [49].

**Figure 4:**
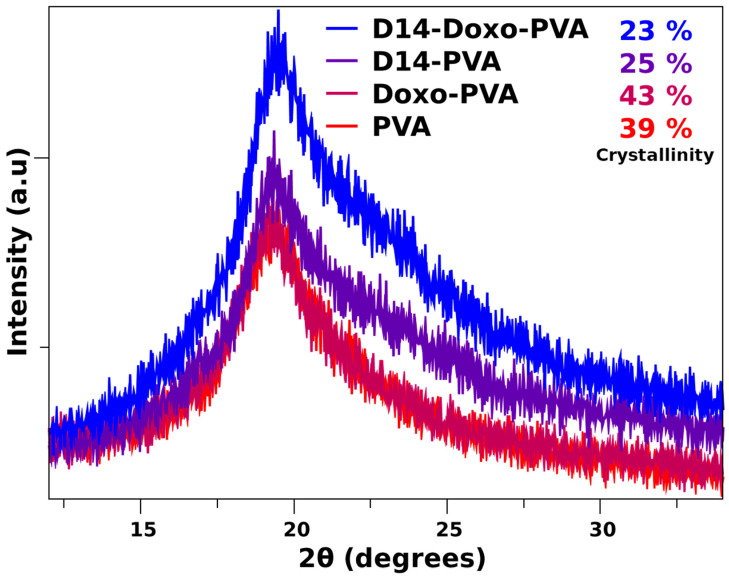
X-ray diffraction pattern of PVA sphere and loaded with D14, doxo and D14-Doxo.

In order to perceive the ordered regions of this hydrogel, XRD analysis (Figure 4) evidenced that the pattern of pure PVA displays a large peak that is typical of its crystallographic reflection and corresponds to the crystalline and semicrystalline nature of PVA [39,50]. In addition, the crystalline regions for specific conjugated structures of doxorubicin and D14 have been reported before, which indicates its major peaks around the same region of pure PVA [51,52]. Nonetheless, deconvolution of the spectra was performed (Appendix A) in order to obtain an amorphous and crystalline region. Within these, it was found that the crystallinity of the composite remains stable when doxo is added, but it is heavily decreased with the addition of D14, and this is further decreased when both drugs are added. This may be related to their inherited microstructure, in which the interaction between these drugs decreased the chain orientation of PVA, whereas they are now conformed within the structure of the drug. This is in agreement with the thermal analysis data, confirming that there is an interaction of the polymeric chains around the drug functional groups.

**Figure 5:**
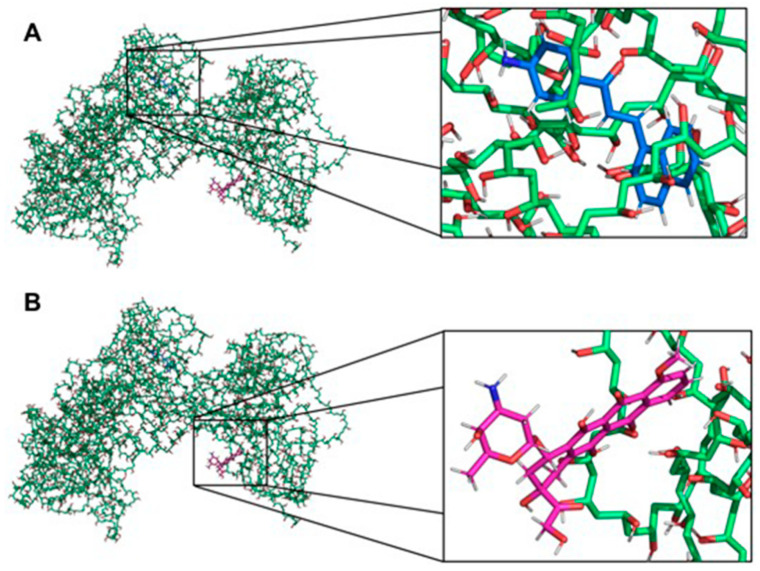
Interaction between the PVA and the drugs. (**A**) Interaction between PVA and D14. The PVA and the drug are presented as sticks highlighted in green and blue, respectively. (**B**) Interaction between PVA and doxo. The PVA and the drug are presented as sticks highlighted in green and pink, respectively. The non-polar hydrogens were removed for better visualization of the interactions. The figures were made with PyMOL software (v.1.1.2, Warren Lyford DeLano, open-source software available at [53].

Aiming to illustrate the chemical interactions between the polymer and the drugs, molecular docking studies were used. A strong interaction of D14 with PVA was observed. As shown in Figure 5A, the D14 molecule binds to the hydrophobic cavity of PVA, confirming the data observed in the experimental results (Figure 2, Figure 3 and Figure 4). However, doxo binds only with a portion of its structure on PVA (Figure 5B), suggesting a weakened interaction, and the major part of the doxo structure keeps interacting with the solvent, probably through hydrogen bonds. Due to the non-polar nature of D14, polar characteristics of doxo and previous experimental studies, these results were expected.

**Figure 6:**
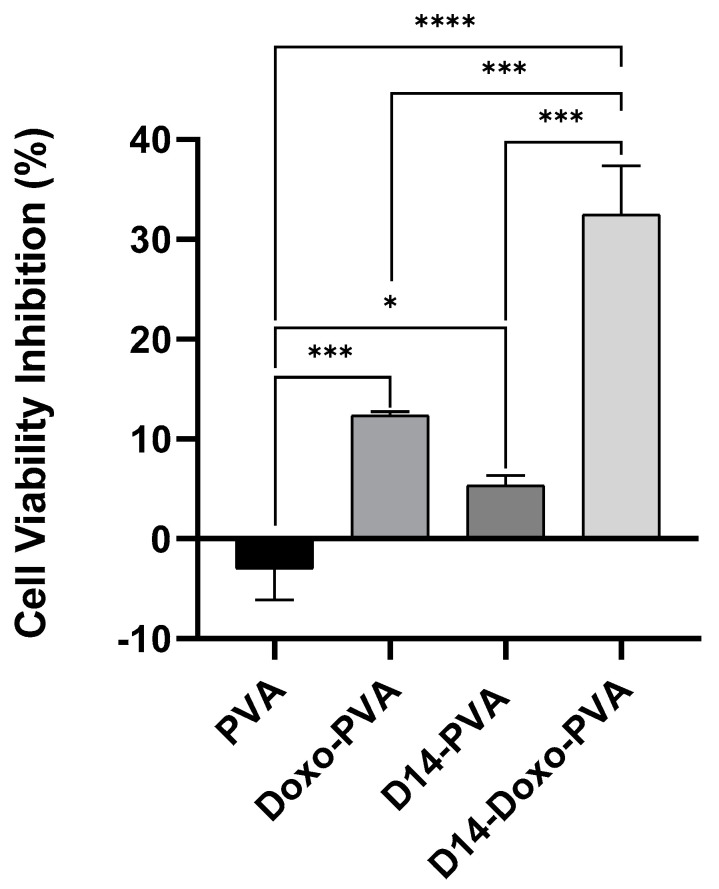
MTT assay to measure cell viability of U2OS cells treated for 24 h with PVA sphere containing only D14 (~8 µg/mL) or doxo (~4 µg/mL), PVA sphere loaded with both drugs D14 + doxo and PVA empty spheres. The cell viability % is obtained by the ratio of the treated cells to the control and data is shown as mean +- standard deviation. Statistical analysis was performed by using one-way ANOVA followed by Tukey post-test and significant results were considered when *, *** < 0.001 and **** < 0.0001.

In order to explore the effects of the PVA spheres loaded with drugs on cell viability, the MTT assay was performed. The results obtained in this assay were consistent with what was expected (Figure 6). Whereas empty PVA spheres did not reduce the cell viability, confirming the non-toxicity of the polymer, PVA spheres loaded with D14 did not present high cytotoxicity against bone cancer cells. This is in agreement with our previous studies, in which we demonstrated that D14 has great potential to inhibit migration and cellular invasion but exhibits low cytotoxicity against osteosarcoma cells [6]. The PVA spheres carrying doxo reduced cell viability by 12.3% (Figure 6).

Previous studies have shown that some chalcones have the capacity to enhance the bioactivity of doxo [7,54]. Moreover, activators of the tumor protein p53 and the activating transcription factor 3 (ATF3) could synergistically improve doxo cytotoxicity in cancer cell lines [14,55]. In previous experiments, our group found that D14 can activate both p53 and ATF3, and this prompted us to hypothesize that D14 could enhance the anticancer effect of doxo. Based on this, PVA spheres were used to simultaneously encapsulate D14 and doxo; then the cytotoxicity of these spheres in osteosarcoma cells was evaluated. As shown in Figure 6, the presence of D14 increased the cytotoxicity of doxo by more than 2.6-fold. Calculating the CDI to evaluate the synergism, a CDI value of 0.49 was obtained, indicating a substantial synergism of the association of D14 and doxo. It is important to consider that when this result was compared to a 24 h treatment with just the drugs, alone and in combination, the cytotoxicity promoted by D14 and doxo was maintained (Appendix A); hence, D14-doxo-loaded spheres could be an interesting option for the treatment of osteosarcoma.

## 4. Conclusions

In this work, a freeze–thaw method was used to develop PVA spheres simultaneously loaded with a hydrophilic drug (doxo) and a hydrophobic drug (D14) for use as a dual DDS. To the best of our knowledge, this manuscript is the first study evolving production and characterization of freeze–thaw PVA spheres loaded with doxo and D14 that maintained the anticancer activity of the drugs. Physical-chemical characterization demonstrated that the drugs were efficiently incorporated within the PVA matrix through physical interactions and weak chemical interactions. The in vitro cytotoxic tests showed that PVA spheres were able to maintain the biologic activity of the drugs, and that the co-delivery of doxo and D14 was superior in terms of cytotoxicity against osteosarcoma compared with doxo or D14 administered alone. This evidences the synergism of the combination of these two compounds. These results highlight the potential application of PVA spheres as a delivery system of the synergistic association from chemotherapeutics drugs to further increase anticancer efficacy.

## Figures and Tables

**Figure A1:**
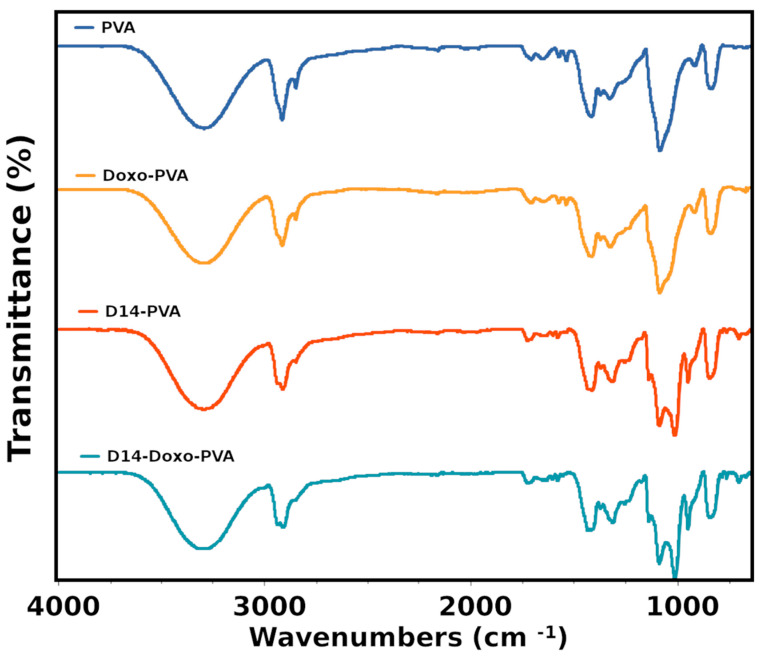
FTIR of the studied samples exhibiting the whole scanned region.

**Figure A2:**
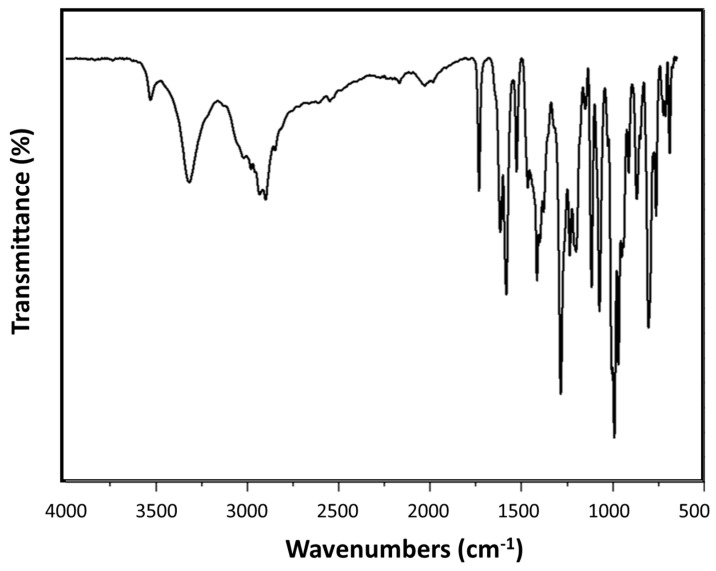
FTIR spectra of doxorubicin detailing a large number of important bands around 1800–500 cm^−1^.

**Figure A3:**
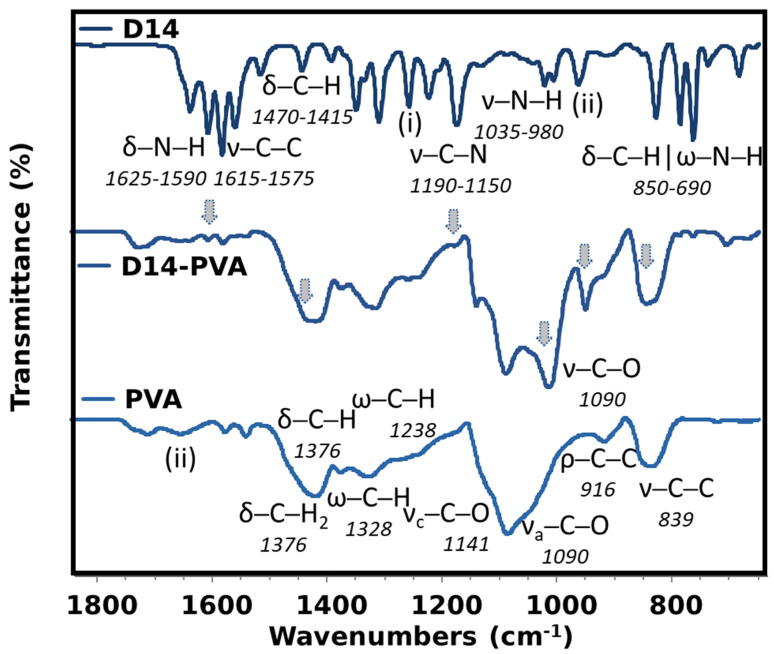
Detailed FTIR spectra of pure D14, PVA and the conjugated D14.

**Figure A4:**
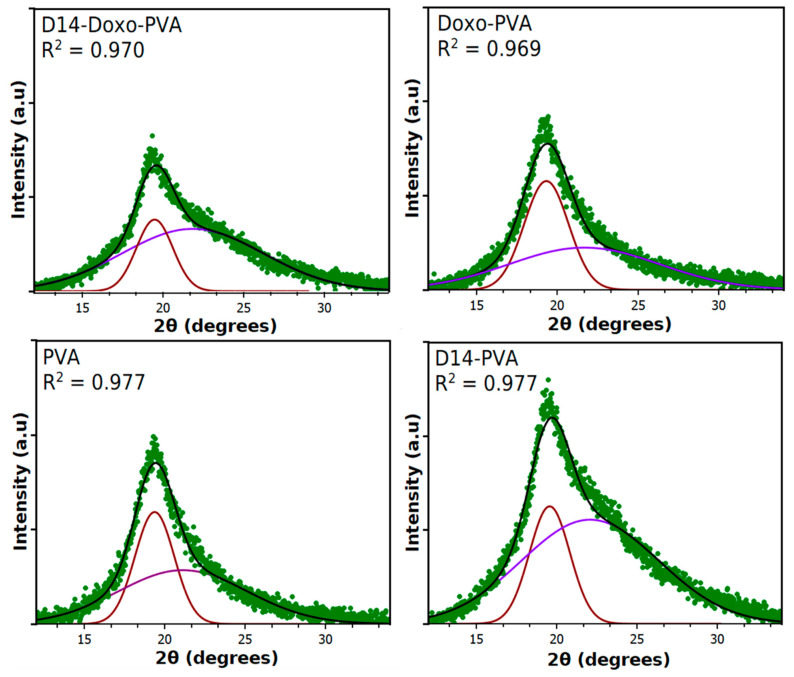
Deconvolution of the PVA majority peak into amorphous and crystallinity peaks in order to calculate the % crystallinity. Only two peaks were used for deconvolution in order to represent an average value of the whole hydrogel.

**Figure A5:**
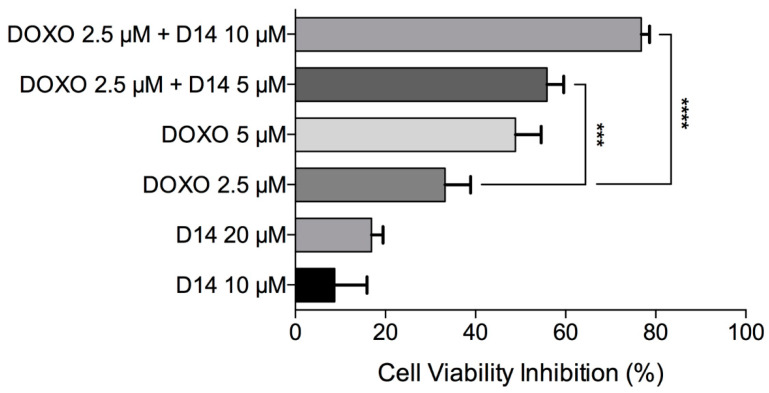
Cell viability inhibition after exposure to doxo, D14 and the combination of both drugs. Data is shown as mean and standard deviation.

## Data Availability

Data will be made available upon request.
